# Explore the gene network regulating the composition of fatty acids in cottonseed

**DOI:** 10.1186/s12870-021-02952-4

**Published:** 2021-04-13

**Authors:** Lihong Ma, Xinqi Cheng, Chuan Wang, Xinyu Zhang, Fei Xue, Yanjun Li, Qianhao Zhu, Jie Sun, Feng Liu

**Affiliations:** 1 Key Laboratory of Oasis Eco-agriculture, College of Agriculture, Shihezi University, Shihezi, 832000 Xinjiang China; 2 CSIRO Agriculture and Food, GPO Box 1700, Canberra, 2601 Australia

**Keywords:** *Gossypium hirsutum*, DEGs, Transcriptomic analysis, Fatty acid components, Co-expression network

## Abstract

**Background:**

Cottonseed is one of the major sources of vegetable oil. Analysis of the dynamic changes of fatty acid components and the genes regulating the composition of fatty acids of cottonseed oil is of great significance for understanding the biological processes underlying biosynthesis of fatty acids and for genetic improving the oil nutritional qualities.

**Results:**

In this study, we investigated the dynamic relationship of 13 fatty acid components at 12 developmental time points of cottonseed (*Gossypium hirsutum* L.) and generated cottonseed transcriptome of the 12 time points. At 5–15 day post anthesis (DPA), the contents of polyunsaturated linolenic acid (C18:3n-3) and saturated stearic acid (C18:0) were higher, while linoleic acid (C18:2n-6) was mainly synthesized after 15 DPA. Using 5 DPA as a reference, 15,647 non-redundant differentially expressed genes were identified in 10–60 DPA cottonseed. Co-expression gene network analysis identified six modules containing 3275 genes significantly associated with middle-late seed developmental stages and enriched with genes related to the linoleic acid metabolic pathway and α-linolenic acid metabolism. Genes (*Gh_D03G0588* and *Gh_A02G1788*) encoding stearoyl-ACP desaturase were identified as hub genes and significantly up-regulated at 25 DPA. They seemed to play a decisive role in determining the ratio of saturated fatty acids to unsaturated fatty acids. *FAD2* genes (*Gh_A13G1850* and *Gh_D13G2238*) were highly expressed at 25–50 DPA, eventually leading to the high content of C18:2n-6 in cottonseed. The content of C18:3n-3 was significantly decreased from 5 DPA (7.44%) to 25 DPA (0.11%) and correlated with the expression characteristics of *Gh_A09G0848* and *Gh_D09G0870*.

**Conclusions:**

These results contribute to our understanding on the relationship between the accumulation pattern of fatty acid components and the expression characteristics of key genes involved in fatty acid biosynthesis during the entire period of cottonseed development.

**Supplementary Information:**

The online version contains supplementary material available at 10.1186/s12870-021-02952-4.

## Background

The quality and nutritional value of plant oils are largely defined by their fatty acid compositions. Because of the low freezing point of saturated fatty acids (SFAs), vegetable oils with a high content of SFAs will coagulate, turbid, semi solidified or even solidified at a lower temperature. From health perspective, SFAs may increase the risk of cardiovascular diseases [1–4]. Polyunsaturated fatty acids (PUFAs), e. g. linoleic acid (C18:2n-6) and linolenic acid (C18:3n-3), are beneficial to human health, but they are easily oxidized and become rancid when exposed to oxygen under general storage conditions [4–7]. Chemical hydrogenation of PUFAs can improve the oxidation stability of oil, but unfortunately, it produces harmful trans fatty acids as by-products [8, 9]. Monounsaturated fatty acid (MUFA) oleic acid (C18:1n-9) is stable and considered to be beneficial to human health [3, 8–12]. Edible oil with a high proportion of C18:1n-9 represents “healthy oil”, such as olive oil, due to low content of SFAs and no need of chemical hydrogenation [13].

Cottonseed kernel contains 25–35% oil and is one of the major sources of edible vegetable oil. Its nutritional components and quality have thus attracted much attention. The major fatty acid components of cottonseed oil are linoleic acid (C18:2n-6), palmitic acid (C16:0), oleic acid (C18:1n-9) and stearic acid (C18:0). Generally, the relative content of C18:1n-9 in cottonseed oil is ~ 15%. Because of its nutritional benefit, increasing the relative content of C18:1n-9 is one of the major targets of genetic improvement of plant oils. In higher plants, C18:1n-9 is converted from C18:1n-9-ACP by one of the acyl-ACP thioesterases, FATA, and is then used as substrate to synthesize C18:2n-6, the most abundant fatty acid component of cottonseed oil, by fatty acid desaturase 2 (FAD2) [14]. Genetically modified plants with high oleic acid (C18:1n-9) content have been developed in several crops, e.g. soybean [15, 16], rapeseed [17, 18], cotton [3, 10, 19] and jatropha [20], by decreasing *FAD2* expression. Changes in fatty acid composition improved the nutritional value but may have unexpected side effects, such as reduced total oil content or seed vigor [3, 8], probably due to the natural compositions of various fatty acids in seeds as a result of natural selection being necessary for seed germination and seedling growth. Composition of seed fatty acids is also closely related to the self-protection and physiological defense mechanisms of offspring [3, 21]. Therefore in-depth analysis of the gene network regulating fatty acid components of cottonseed oil is of great significance for their genetic modification to meet the needs of both human health and the fitness of cotton plants.

In this study, we first investigated the dynamic changes of fatty acid components in the developing seeds of an Upland cotton (*Gossypium hirsutum* L.) cultivar Xinluzao 33 and generated transcriptomes of developing seeds at multiple developmental time points. We then analyzed the expression profiles of the key genes involved in fatty acid biosynthesis and performed gene network analysis using differentially expressed genes (DEGs). The results deepened our understanding of the genes associated with the fatty acid metabolic network and the temporal regulation of cottonseed fatty acid components, and provided gene expression information that may be helpful for identification of targets for improvement of cottonseed oil quality.

## Results

### Dynamic profiles of fatty acids during cottonseed development

To investigate the dynamic changes of fatty acid components during cottonseed development, the relative contents of fatty acid components were determined by GC-MS. In total, 13 fatty acid components at 12 developmental time points, separated into three stages, i.e. early (5–15 DPA), middle (20–35 DPA) and late (40–60 DPA), were determined. In the mature cotton seeds, the main fatty acid components were polyunsaturated linoleic acid (C18:2n-6, 57.62%), monounsaturated oleic acid (C18:1n-9, 15.59%), saturated palmitic acid (C16:0, 22.36%) and stearic acid (C18:0, 2.31%), all other components accounted for less than 1% of the total oil content. The relative content of C18:2n-6 gradually increased from 5 to 15 DPA, significantly increased after 15 DPA, reached a higher level at 25 DPA, and tended to be stable after that (Table 1). The highest relative content of C18:1n-9 was observed at 5 DPA (23.42%), then decreased significantly in 10 to 15 DPA seeds and increased again from 15 to 20 DPA seeds, and remained relatively constant afterwards until maturity. Saturated C16:0 was the second highest fatty acid component in the mature cottonseeds with its maximum relative percentage (44.23%) observed at 15 DPA and a significant reduction from 15 (28.35%) to 20 DPA (23.45%). C18:0 had a similar dynamic change profile as C16:0, with its maximum relative content (13.75%) observed at 15 DPA, then rapidly decreased to 5.29% at 20 DPA and finally to 2.31% in the mature seeds. Obviously, it seems that C16:0 and C18:0 synchronously accumulated mainly during the early (5–15 DPA) and middle (20–35 DPA) stages of seed development. It should be noted that the highest relative content (7.44%) of linolenic acid C18:3n-3 was observed in 5 DPA seeds, decreased to 3.12% at 10 DPA and further down to 0.55% at 20 DPA, and was one of the least in the mature seeds (Table 1). There was a significantly negative correlation between the relative content of C18:2n-6 and C18:3n-3. During development of cottonseeds, the relative content of myristic acid (C14:0), palmitoleic acid (C16:1), arachidic acid (C20:0), behenic acid (C22:0) and lignoceric acid (C24:0) was no more than ~ 2% with a relatively high percentage observed in the early stage. With the development of cottonseeds, the content of these fatty acids gradually decreased from middle to late (20–60 DPA) stages and remained at a very low level (< 1%) in mature seeds. The relative content of heptadecanoic acid (C17:0) was constantly low and even not detected at 20–25 DPA. Both trans-9-octadecenoic acid (C18:1n-9 t) and all-trans-9,12-octadecadienoic acid (C18:2n-6tt) were only detectable at 5–15 DPA (Table 1).

**Table 1:** Percentage of fatty acid components in oil from 5 to 60 DPA cottonseeds

Fatty acid components	5 DPA	10 DPA	15 DPA	20 DPA	25 DPA	30 DPA	35 DPA	40 DPA	45 DPA	50 DPA	55 DPA	60 DPA
C14:0	1.35 ± 0.12	1.48 ± 0.05	1.76 ± 0.02	2.12 ± 0.09	0.68 ± 0.02	0.70 ± 0.01	0.79 ± 0.12	0.70 ± 0.04	0.58 ± 0.02	0.58 ± 0.06	0.58 ± 0.03	0.80 ± 0.04
C16:0	33.96 ± 1.66	42.36 ± 1.40	44.23 ± 0.69	28.35 ± 0.85	23.35 ± 0.09	24.13 ± 0.35	27.07 ± 0.37	24.46 ± 0.30	22.39 ± 0.42	21.16 ± 0.40	21.57 ± 0.39	22.35 ± 0.41
C17:0	0.20 ± 0.02	0.22 ± 0.02	0.26 ± 0.02	–	–	0.11 ± 0.01	0.10 ± 0.01	0.11 ± 0.01	0.09 ± 0.04	0.09 ± 0.01	0.10 ± 0.02	0.11 ± 0.02
C18:0	9.02 ± 0.49	13.41 ± 0.05	13.75 ± 0.82	5.29 ± 0.17	2.28 ± 0.09	1.93 ± 0.10	2.10 ± 0.13	2.30 ± 0.07	2.11 ± 0.12	2.14 ± 0.10	2.19 ± 0.09	2.31 ± 0.05
C20:0	0.93 ± 0.07	0.81 ± 0.19	0.74 ± 0.03	0.85 ± 0.02	0.42 ± 0.02	0.33 ± 0.04	0.05 ± 0.01	0.27 ± 0.01	0.24 ± 0.02	0.22 ± 0.10	0.23 ± 0.07	0.30 ± 0.07
C22:0	1.27 ± 0.05	0.74 ± 0.00	0.56 ± 0.15	0.59 ± 0.02	0.29 ± 0.01	0.22 ± 0.01	0.10 ± 0.04	0.18 ± 0.01	0.14 ± 0.03	0.14 ± 0.02	0.14 ± 0.04	0.21 ± 0.04
C24:0	0.66 ± 0.00	0.34 ± 0.05	0.21 ± 0.01	0.38 ± 0.01	0.16 ± 0.01	0.09 ± 0.01	0.79 ± 0.02	0.11 ± 0.01	0.08 ± 0.00	0.07 ± 0.01	0.08 ± 0.01	0.10 ± 0.00
SFAs	47.39 ± 2.32	59.36 ± 1.69	61.51 ± 1.57	37.58 ± 1.09	27.18 ± 0.63	27.51 ± 1.01	31.00 ± 1.02	28.13 ± 0.66	25.63 ± 0.79	24.40 ± 0.66	24.89 ± 0.71	26.18 ± 0.93
C16:1	1.05 ± 0.05	1.30 ± 0.08	0.93 ± 0.06	0.38 ± 0.02	0.61 ± 0.01	0.49 ± 0.02	0.43 ± 0.05	0.53 ± 0.02	0.62 ± 0.08	0.58 ± 0.05	0.64 ± 0.02	0.58 ± 0.04
C18:1n-9	23.42 ± 0.12	9.87 ± 0.12	8.77 ± 0.57	15.08 ± 0.69	13.08 ± 0.45	12.28 ± 0.87	13.90 ± 0.63	13.17 ± 0.32	12.97 ± 0.54	15.84 ± 0.61	13.54 ± 0.68	15.59 ± 0.94
C18:1n-9 t	0.10 ± 0.01	0.17 ± 0.01	0.10 ± 0.00	–	–	–	–	–	–	–	–	–
C18:2n-6	20.32 ± 1.38	22.62 ± 0.82	27.46 ± 0.14	46.41 ± 0.32	58.96 ± 0.40	59.62 ± 1.04	54.56 ± 0.46	58.11 ± 0.07	60.56 ± 0.24	59.16 ± 0.82	60.91 ± 0.61	57.62 ± 0.62
C18:2n-6tt	0.27 ± 0.00	0.18 ± 0.01	0.06 ± 0.00	–	–	–	–	–	–	–	–	–
C18:3n-3	7.44 ± 0.13	3.12 ± 0.40	1.16 ± 0.35	0.55 ± 0.03	0.11 ± 0.01	0.09 ± 0.01	0.06 ± 0.00	0.05 ± 0.00	0.03 ± 0.01	0.02 ± 0.01	0.01 ± 0.00	0.02 ± 0.00
UFAs	52.6 ± 1.57	37.26 ± 1.32	38.48 ± 1.09	62.42 ± 1.34	72.76 ± 1.56	72.48 ± 2.03	69.35 ± 1.75	71.86 ± 1.33	74.18 ± 2.09	75.60 ± 2.39	75.10 ± 2.31	73.81 ± 1.89

The percentage of each fatty acid component is the average of three repeated experiments; “--” indicates not detected. *SFAs* saturated fatty acids, *UFAs* unsaturated fatty acids. Data were presented as the mean ± SD of three replications

### Transcriptome analysis

To understand how the dynamic changes in fatty acid components during cottonseed development are regulated, we generated seed transcriptome profiles using RNA-Sequencing (RNA-seq). RNA-seq libraries were generated from 12 different developmental time points (5, 10, 15, 20, 25, 30, 35, 40, 45, 50, 55 and 60 DPA) and sequenced. A total of ~ 599 million raw reads were generated. The average Q30 of the reads from all samples was 94.04% and the read GC content was 44.66%. Raw reads were filtered to remove low quality ones and a total of 576,668,718 clean reads were finally used in alignment. Overall, from 93.35 to 96.38% of the clean reads could be aligned to the reference genome with 87.80–90.09% of them being uniquely aligned (Table S3). These results suggested that the RNA-seq data were high quality and suitable for further analyses.

We identified genes differentially expressed at 10–60 DPA seeds using 5 DPA seeds as a control. From 10 DPA to 20 DPA, the number of DEGs increased from 396 to 2451. The number of DEGs at 25, 30 and 35 DPA three middle developmental time points was similar and started to increase at 40 DPA with a significant increase observed at 55 and 60 DPA (Fig. S2A). Regarding the number of up- and down-regulated DEGs, two transition points were observed at 10–15 DPA and 40–45 DPA. There were more down-regulated DEGs than up-regulated ones at 10 DPA and 45–60 DPA, but more up-regulated DEGs than down-regulated ones at 15–40 DPA (Fig. S2A). The period from 10 to 30 DPA is critical for oil accumulation. We further compared DEGs at 10, 20 and 30 DPA three time points. In the comparison group of 20 DPA vs 10 DPA, 30 DPA vs 20 DPA and 30 DPA vs 10 DPA, 1268, 1607, and 2517 DEGs were up-regulated, and 430, 1030 and 1302 DEGs were down-regulated, respectively. Among these DEGs, 501, 667 and 1413 were unique to each comparison, respectively, and 113 genes showed differential expression in all three comparisons (Fig. S2B).

### Expression profiles of the genes involved in biosynthesis of fatty acids during cottonseed development

**Fig. 1:**
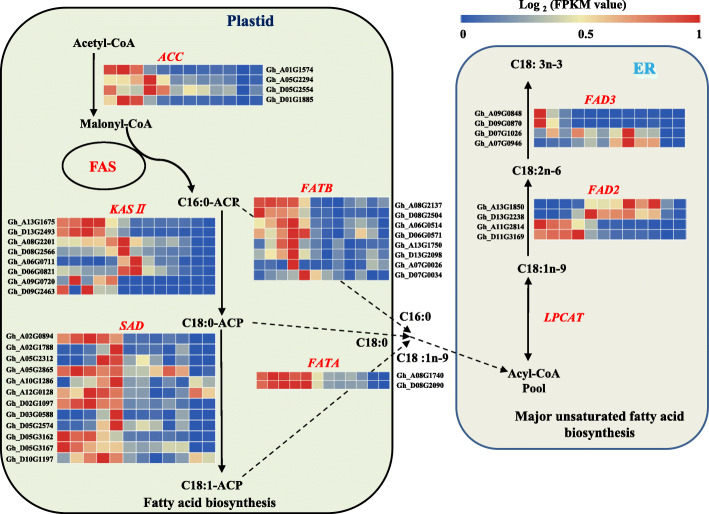
Gene expression network of oil accumulation in developing cottonseed. The expression patterns of each differentially expressed unigene was shown on the relative log2 (FPKM value) from left to right is 0 ~ 60 DPA. *ACC*, acetyl-CoA carboxylase gene; FAS, Fatty Acid Synthase; *KASII*, β-ketoacyl ACP synthase II gene; *SAD*, stearoyl-ACP desaturase gene; *FATA/B*, fatty acyl-ACP thioesterase A/B gene; *FAD2*, fatty acid desaturase 2 gene; *FAD*3, fatty acid desaturase 3 gene; C16:0, palmitic acid; C18:0, stearic acid; C18:1n-9, oleic acid; C18:2n-6, linoleic acid; C18:3n-3, α-linolenic acid. This model was modified based on references of  Zhao et al. (2018) [22]

The de novo biosynthesis of fatty acids takes place mainly in the plastid. We first investigated the expression dynamics of the major genes involved in fatty acid biosynthesis (Fig. 1). The first step of fatty acid biosynthesis is the formation of malonyl coenzyme by acetyl-CoA carboxylase (ACC), which is a key rate-limiting enzyme in fatty acid biosynthesis [23] and has a great impact on the final level of seed fatty acid content. The cotton genome contains two pairs of homoeologous *ACC* genes (*Gh_A01G1574* and *Gh_D01G1885*, *Gh_A05G2294* and *Gh_D05G2554*). All four genes had a higher expression level in the early to middle developmental stages than in the late stages but the two pair homoeologs had a different profile, with *Gh_A01G1574* and *Gh_D01G1885* being mainly expressed in 5–15 DPA seeds while *Gh_A05G2294* and *Gh_D05G2554* being mainly expressed in 20–25 DPA seeds. Malonyl-CoA is catalyzed by fatty acid synthase (FAS) to produce 16:0 carbon palmitoyl-ACP via the recurring reactions. The elongation of the 16-carbon palmitoyl-ACP to the 18-carbon stearoyl-ACP is catalyzed by β-ketoacyl-ACP synthase II [24]. Cotton has four pairs of homoeologous genes encoding β-ketoacyl-ACP synthase II (KASII). They are *Gh_A13G1675* and *Gh_D13G2493*, *Gh_A08G2201* and *Gh_D08G2566*, *Gh_A06G0711* and *Gh_D06G0821*, and *Gh_A09G0720* and *Gh_D09G2463*. *Gh_A13G1675* and *Gh_D13G2493* were mainly expressed in 5–15 DPA seeds, *Gh_A08G2201* and *Gh_D08G2566* were mainly expressed in 20–25 DPA seeds, and *Gh_A06G0711* and *Gh_D06G0821* had their highest expression levels observed at 30 DPA. These results suggest that *Gh_A13G1675* and *Gh_D13G2493* might be the major genes contributing to the conversion from C16:0-ACP to C18:0-ACP in cottonseeds. C18:0-ACP is desaturated by ^Δ9^-stearoyl-ACP desaturase (SAD) to form monounsaturated C18:1n-9-ACP. Twelve *SAD* genes (*Gh_A02G0894, Gh_D02G1097, Gh_A02G1788, Gh_D03G0588, Gh_A05G2865, Gh_D05G3162, Gh_A10G1286, Gh_D10G1197, Gh_A05G2312, Gh_D05G2574, Gh_A12G0128* and *Gh_D05G3167*) were identified in the cotton genome. Most had a higher expression level in 5–25 DPA seeds than in 30–60 DPA seeds, with the exception of two pairs of homoeologs (*Gh_A02G1788* and *Gh_D03G0588*, *Gh_A05G2312* and *Gh_D05G2574*), which had high expression levels at 20–25 DPA (Fig. 1).

In plants, the acyl group from acyl-ACP can be hydrolyzed by two different classes of acyl-ACP thioesterases (FATs), FATA and FATB, to release free fatty acids. During cottonseed development, one pair of homoeologous *FATA* gene (*Gh_A08G1740* and *Gh_D08G2090*) was highly expressed, particularly in 5–25 DPA seeds, and three of the four pairs of homoeologous *FATB* (*Gh_A08G2137* and *Gh_D08G2504*, *Gh_A06G0514* and *Gh_D06G0571*, *Gh_A13G1750* and *Gh_D13G2098*, and *Gh_A07G0026* and *Gh_D07G0034*) were also mainly expressed in 5–25 DPA seeds. The specificity of FATs is the major determinant of the chain length and the level of saturated fatty acids (SFAs) found in most plant tissues [25]. It had been shown that FATB displayed high catalytic efficiencies towards 16:0-ACP, whereas FATA had a high activity with oleoyl-ACP and a low activity with 16:0-ACP (~ 75-fold lower than FATB) [25]. At the early developmental stage of cottonseed, the expression levels of *KASII* and *FATA* were high, resulted in a higher accumulation of C18:0 in 0–15 DPA seeds. However, C18:0-ACP is also the substrate for synthesis of C18:1n-9-ACP. Meanwhile, the expression level of *FATBs* was significantly higher than that of *FATAs* at the same developmental stage during cottonseed development, particularly in 15–25 DPA seeds, consequently, the accumulation of C16:0 was much greater than that of C18:0 in cottonseed, and C16:0 being the major SFA in mature cottonseeds.

The major polyunsaturated fatty acid (PUFA) in cottonseed is synthesized by fatty acid desaturase 2 (FAD2, C18:1n-9 to C18:2n-6 desaturation) and polyunsaturated linolenic acid (C18:3n-3) is desaturated from C18:2n-6 by fatty acid desaturase 3 (FAD3). These reactions take place in the endoplasmic reticulum (ER). Two pairs of homoeologous *GhFAD2* (*Gh_A13G1850* and *Gh_D13G2238*, *Gh_A11G2814* and *Gh_D11G3169*) were expressed in developing cottonseeds with different expression patterns. *Gh_A13G1850* and *Gh_D13G2238*, named as *GhFAD2–1* [26], were mainly expressed in 25–50 DPA seeds, while *Gh_A11G2814* and *Gh_D11G3169* were mainly expressed in 5–20 DPA seeds. The relative content of C18:2n-6 increased significantly from 15 to 25 DPA and remained high afterwards, overlapping with the period of high expression of *Gh_A13G1850* and *Gh_D13G2238,* indicating that these two homoeologs are the major contributors of C18:2n-6 biosynthesis, consistent with previous results [26, 27]. *GhFAD3* are encoded by two pairs of homoeologous genes (*Gh_A09G0848* and *Gh_D09G0870*, *Gh_A07G0946* and *Gh_D07G1026*). These two pairs also had a different expression pattern in developing cottonseed. *Gh_A09G0848* and *Gh_D09G0870* had a relatively high expression level in 5 to 15 DPA seeds, particular in 5 DPA. After 20 DPA, their expression level decreased significantly and was almost undetectable in mature cottonseed. In contrast, expression of *Gh_A07G0946* and *Gh_D07G1026* was observed from 5 to 50 DPA, with the highest expression level observed in 40 DPA seeds. C18:3n-3 was apparently accumulated in the early stage of seed development and reached its peak (7.44%) at 5 DPA. The relative content of C18:3n-3 was significantly decreased from 1.16% at 15 DPA to 0.11% at 25 DPA and remained very low afterwards. These results indicate that the content of C18:3n-3 in cottonseed was determined by *Gh_A09G0848* and *Gh_D09G0870* rather than *Gh_A07G0946* and *Gh_D07G1026*.

### Confirmation of DEGs by qRT-PCR analysis

**Fig. 2:**
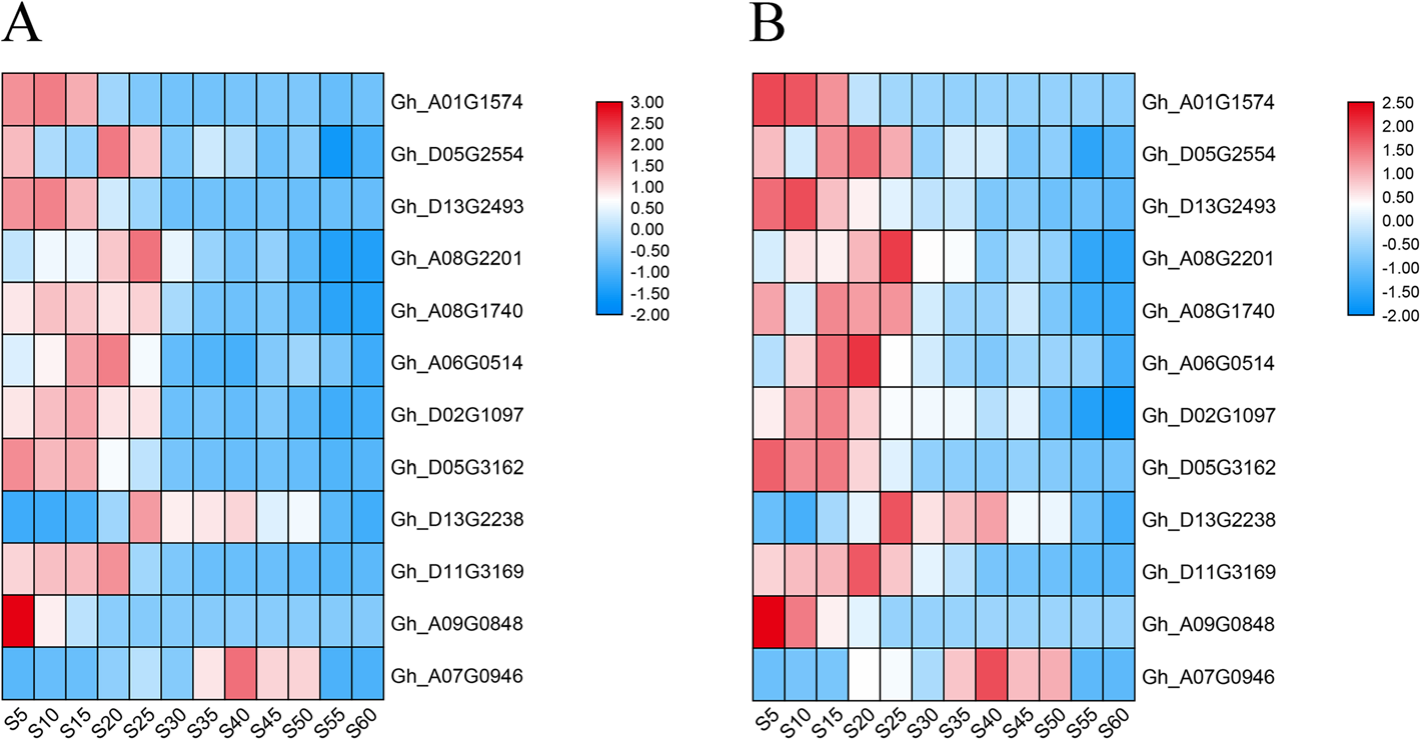
Heatmap showing the relative expression level of the 12 selected genes at the 12 cottonseed developmental stages determined by RNA-seq analysis (**a**) and qRT-PCR (**b**). The selected genes include: *ACC* (*Gh_A01G1574* and *Gh_D05G2554*), *KASII* (*Gh_D13G2493*and *Gh_A08G2201*), *FATA* (*Gh_A08G1740*), *FATB* (*Gh_A06G0514*), *SAD* (*Gh_D02G1097* and *Gh_D05G3162*), *FAD2* (*Gh_D13G2238* and *Gh_D11G3169*), *FAD3* (*Gh_A09G0848* and *Gh_A07G0946*). S 5, S10, S15, S20, S25, S30, S35, S40, S45, S50, S55 and S60 represent 5, 10, 15, 20, 25, 30, 35, 40, 45, 50, 55 and 60 DPA, respectively

To validate the RNA-seq results and identified DEGs, 12 genes related to fatty acid synthesis, including 6 genes related to fatty acid desaturation, were selected for confirmation by qRT-PCR. The relative expression levels and expression tendency of the selected genes determined by qRT-PCR were highly consistent with that of RNA-seq (Fig. 2), indicating highly reliable RNA-seq results.

### Gene ontology analysis of DEGs

**Fig. 3:**
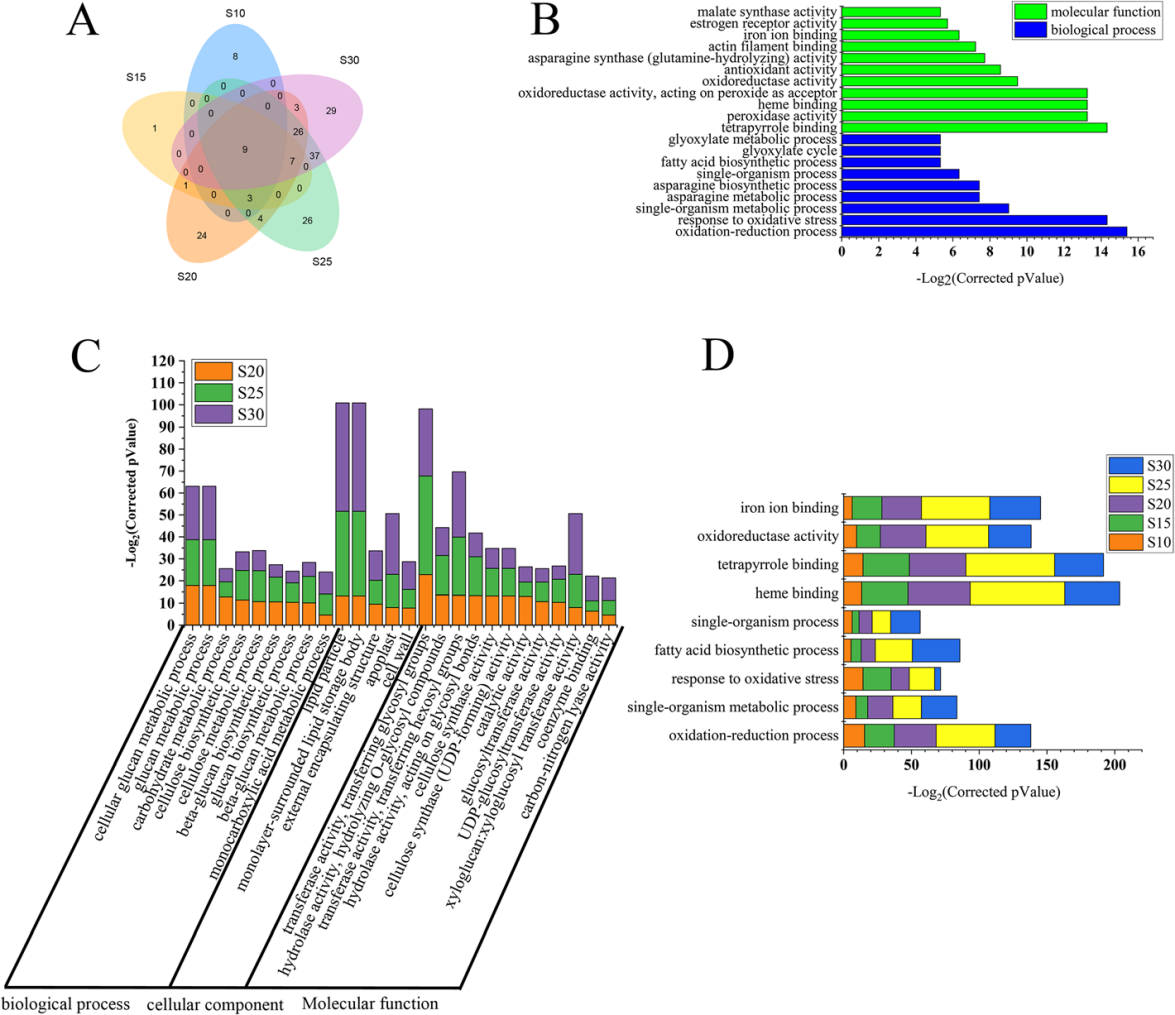
Gene ontology classification of DEGs at various developmental stages of cottonseed. **a.** Venn diagram showing the number of enriched GO terms at the six cottonseed developmental stages. **b.** Analysis of GO enrichment at 10 DPA. **c.** Analysis of GO enrichment at 20, 25 and 30 DPA. **d.** Analysis of GO enrichment at 10, 15, 20, 25 and 30 DPA. S10, S15, S20, S25 and S30 represent 10, 15, 20, 25 and 30 DPA, respectively

To get insights into the biological function of the DEGs, we performed Gene Ontology (GO) analysis using the genes differentially expressed between 5 DPA and other time points. In total, 15,647 non-redundant DEGs were identified and they were enriched with 308 GO terms (Table S4). The relative content of each fatty acid component changed significantly from 5 to 30 DPA and tended to be relatively stable after 30 DPA. The 11,645 non-redundant DEGs identified in 10–30 DPA were enriched with 178 GO terms. Individually, 20, 21, 77, 112 and 111 GO terms were enriched at 10, 15, 20, 25 and 30 DPA, respectively, with 8, 1, 24, 26 and 29 GO terms unique to each time point (Fig. 3a). The number of enriched GO items was significantly fewer at 10 and 15 DPA than at other time points (Fig. 3a, b). Nine commonly enriched GO terms were found in DEGs from 10 to 30 DPA and were related to fatty acid biosynthesis and redox-related process (Fig. 3d). In addition to these GO terms enriched in all time points, 1, 4 and 37 commonly enriched GO terms were found between 15 and 20 DPA, between 20 and 25 DPA, and between 25 and 30 DPA, respectively (Fig. 3a), and 26 GO items were commonly enriched in 20, 25 and 30 DPA three time points (Fig. 3c, Table S5). In 35–60 DPA, 34 GO terms were significantly enriched. In addition, fatty acid synthase activity, fatty acid metabolism and fatty acid biosynthesis processes were the commonly enriched GO items in 35–60 DPA (Table S6).

Through GO analysis of DEGs among different groups, it was found that, in terms of biological processes, these DEGs were mainly related to fatty acids, lipids and other metabolic processes; regarding cell components, they were involved in cell parts and membranes; and their main molecular functions were binding, splitting and catalytic activity. These results suggest that fatty acid synthesis and metabolism-related pathways are mainly enriched in the early stages of cottonseed development, while genes related to cellular component and molecular functions were enriched in the middle development stages.

### KEGG pathways of DEGs

**Fig. 4:**
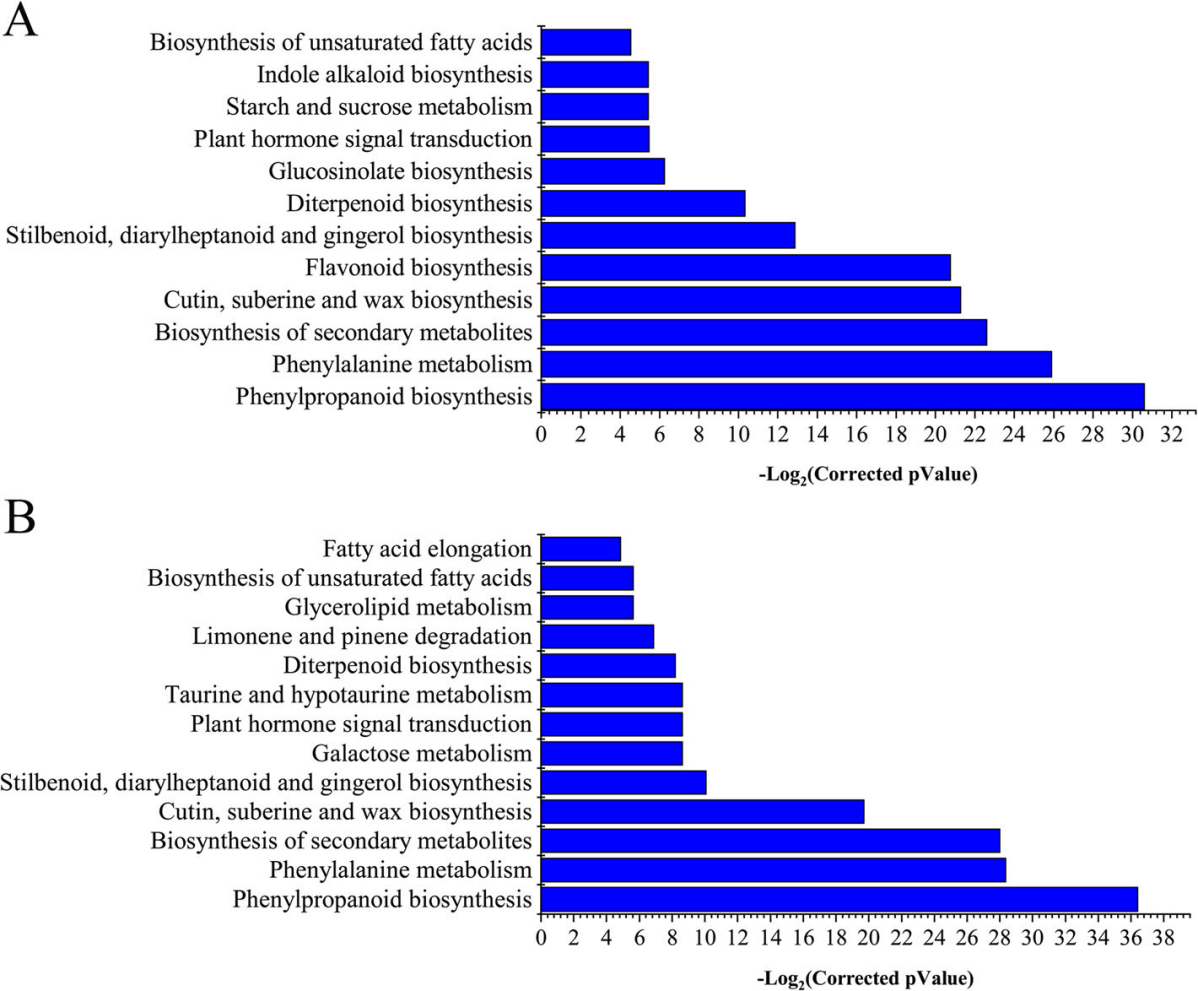
KEGG analysis of DEGs associated with cottonseed developmental at 20 and 25 DPA. **a.** KEGG categories of DEGs associated at 20 DPA. **b.** KEGGs categories of DEGs at 25 DPA. The vertical axes show logarithm (−log_2_) of the corrected *P* value

Pathway analysis was further performed to determine the functions of DEGs. A total of 122 enriched KEGG pathways were found for the 15,647 non-redundant DEGs from 10 to 60 DPA (Table S7). Many significantly changed pathways were related to metabolism of fatty acid elongation, biosynthesis of unsaturated fatty acids, and α-linolenic acid metabolism, which are associated with the synthesis of fatty acid components (Table S8). Generally, C18:3n-3 was mainly synthesized at the early stage (around 5 DPA) of seed development. The enriched KEGG pathways at 10 DPA included those involved in metabolism of phenylalanine, alanine, aspartate and glutamate, and biosynthesis of phenylpropanoid, monoterpenoid, glucosinolate and cutin, suberine and wax. At 20 DPA, pathways related to biosynthesis of flavonoid, diterpenoid, and unsaturated fatty acids were enriched (Fig. 4a, Table S8). *FAD2* (*Gh_D13G2238* and *Gh_A13G1850*) and *FAD3* (*Gh_A09G0848* and *Gh_D09G0870*) were components of these pathways (Fig. S3B). The pathways enriched at 25 DPA were also related to biosynthesis of unsaturated fatty acids and elongation of fatty acids (Fig. 4b, Table S8). Among the 13 DEGs in the fatty acid extension pathway, 12 encodes 3-ketoyl-CoA synthetase (Fig. S3C). The enriched pathways at 30 DPA were similar to that at 25 DPA. We found a remarkable feature that the elongation of fatty acid pathway was enriched in 35, 40, 45, and 50 DPA cottonseed, while the fatty acid degradation pathway was enriched in 60 DPA (Table S9), which might explain why the relative content of the fatty acids tended to be stabilized at the late development stages.

### Gene network analysis with WGCNA

**Fig. 5:**
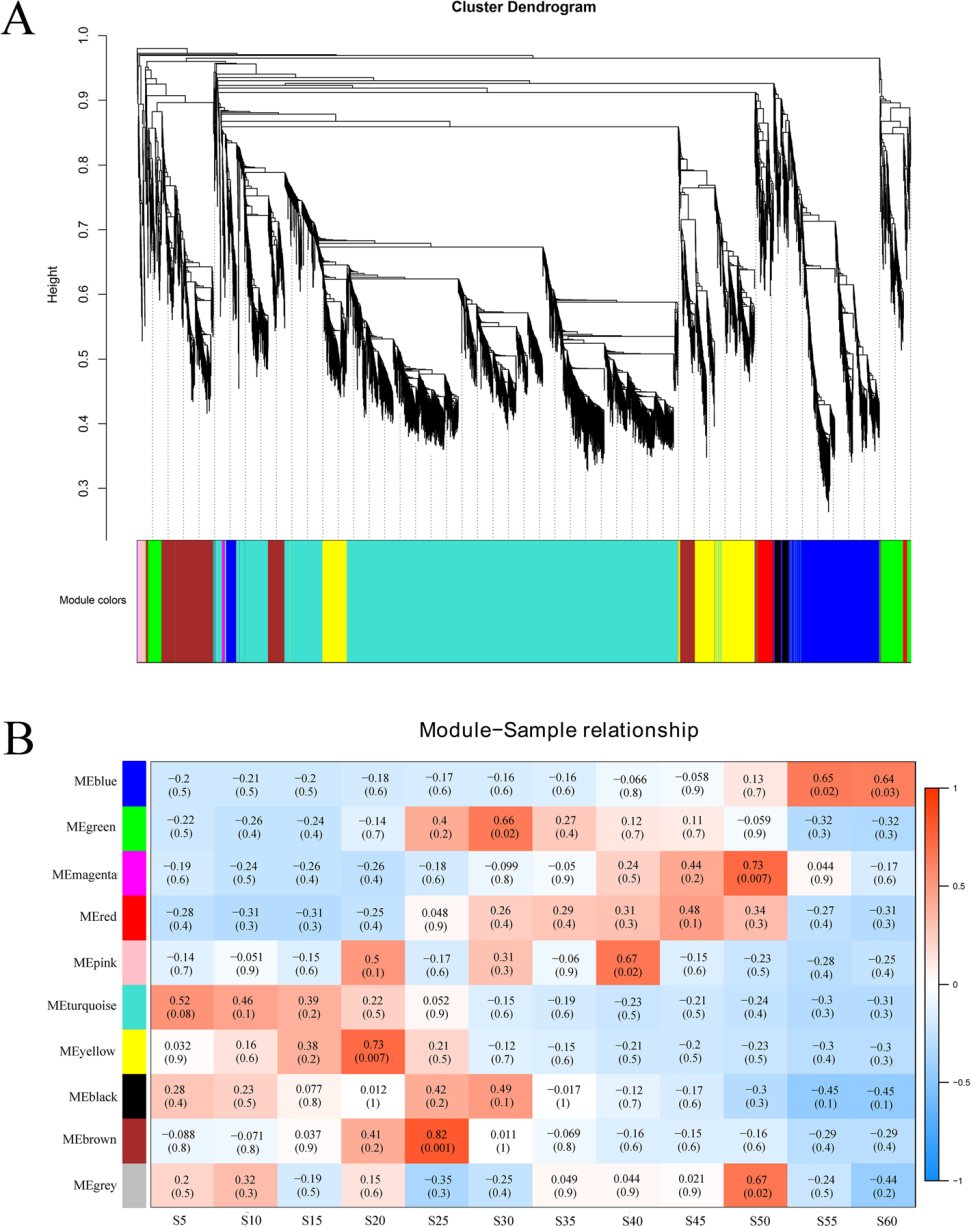
WGCNA of DEGs at different cottonseed developmental stages. **a** Hierarchical cluster tree showing co-expression modules identified by WGCNA. The major tree includes 10 modules according to calculation of eigengenes; each module is highlighted in a designated colour. **b** Module-sample relationships. Each row corresponds to a different module showing on the left side, each column represents a sample, and the correlation coefficient and evalue of each sample-module relationship are displayed. Red represents high expression, and blue represents low expression. S5, S10, S15, S20, S25, S30, S35, S40, S45, S50, S55 and S60 represent 5, 10, 15, 20, 25, 30, 35, 40, 45, 50, 55 and 60 DPA, respectively

WGCNA analysis was performed to obtain a comprehensive understanding the gene network regulating fatty acid synthesis during cottonseed development. The 15,647 non-redundant DEGs identified between 5 DPA and other time points were used in the analysis and a total of 10 gene modules associated with the specific expression profiles of different samples were identified (Fig. 5a). Among these 10 modules, 6 (yellow, brown, green, pink, magenta and blue) were significantly associated with middle and/or late stages of cottonseed development, and no gene module was significantly associated with the early developmental stage (5–15 DPA) although the turquoise module was marginally significant at 5 DPA (Fig. 5b). The yellow module with 861 genes was highly associated with 20 DPA. The brown (870 genes) module was significantly associated with 25 DPA. The green (400 genes) and pink (74 genes) modules were significantly associated with 30 DPA and 40 DPA, respectively. There were 33 genes in the magenta module that was significantly associated with 50 DPA, and 1037 genes in the blue module that was significantly associated with 55 DPA (Fig. 5b). KEGG analysis was performed on 3275 DEGs from these six modules. The modules of yellow, brown, pink and magenta were all enriched with genes involved in the α-linolenic acid metabolism. The relative content of C18:3n-3 was relatively high in 5 DPA seeds (7.44%) and reduced significantly to 0.55% in 20 DPA seeds (Table 1), consistent with significant down-regulation of *GhFAD3* (*Gh_A09G0848* and *Gh_D09G0870*) at 20 DPA (Fig. 1) and C18:3n-3 being a precursor substance for production of jasmonic acid and oxyacids as genes of the α-linolenic acid metabolism pathway involved in biosynthesis of jasmonic acid and oxyacids were up-regulated at 20 DPA (Table S10). In addition, at 25 DPA, the brown module was also related to fatty acid metabolism and biosynthetic pathways; both the pink module and the blue module were enriched with genes involved in the linoleic acid metabolic pathway (Table S10).

### Co-expression gene networks and hub genes associated with fatty acid synthesis

**Fig. 6:**
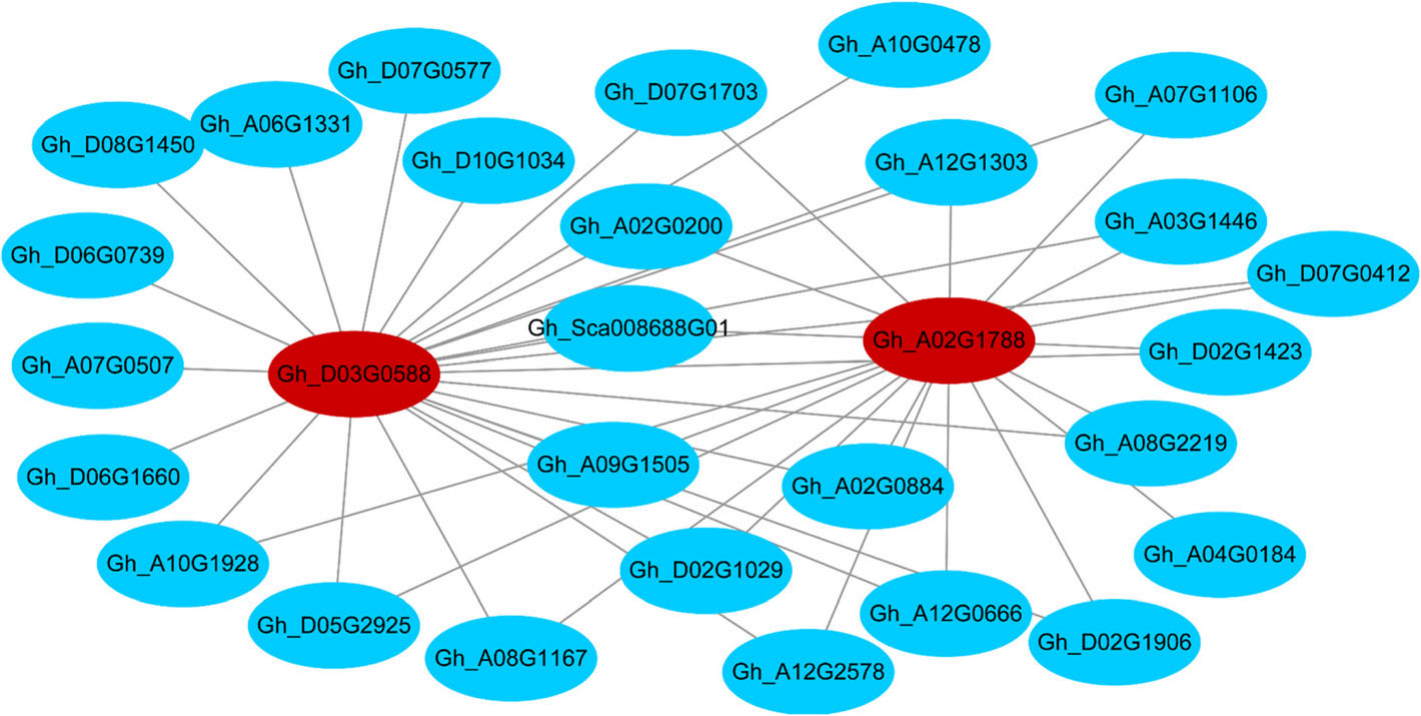
Co-expression network analysis. Red circle represented the hub genes. Co-expression network analysis of the brown module

According to the changes of the relative content of fatty acids during cottonseed development and the results of gene network analysis, the yellow and brown modules were chosen for co-expression analysis to identify the hub genes of the modules. Based on the criteria of eigengene-based connectivity (*K*_*ME*_) value ≥0.89 and edge weight value ≥0.5, 29 genes were found to be co-expressed in the brown module. *Gh_D03G0588* and *Gh_A02G1788* encoding SAD were identified to be the hub genes of the brown module (Fig. 6). At 25 DPA, *Gh_D03G0588* and *Gh_A02G1788* were significantly up-regulated (Fig. 6, Table S11). At this time point, the relative content of C18:0 decreased significantly, indicating that most C18:0-ACP might has been used in synthesis of C18:1n-9-ACP due to significantly increased expression level of *SAD* genes. However, the relative content of C18:1n-9 did not increase significantly, indicating rapid desaturation and conversion of C18:1n-9-ACP to C18:1n-9 and finally C18:2n-6, the major unsaturated fatty acid in cottonseed (Table 1).

## Discussion

Cotton is not only the most important fiber crop, but also an important oil crop. Our results showed that the content of SFAs in cottonseed oil is relatively high (over 20% of C16:0). It means that long-term consumption of cottonseed oil might increase the incidence of cardiovascular diseases. PUFAs that are easy to be oxidized and unstable under the conditions of light and high temperature are also relatively high (up to 60%), while the content of C18:1n-9, a fatty acid being stable and beneficial to human health, is only 15.6% in cottonseed oil, far lower than 75.7% in olive oil [28], 55.9–72.0% in rapeseed oil [29], 28.0% in sunflower oil [2], 41.5% in sesame oil [2], 42.9% in palm oil and 29.1% in soybean oil [28]. Therefore, it has great practical value to enhance effectively the content of C18:1n-9 in cottonseed to improve the nutritional quality of cottonseed oil.

Understanding plant lipid metabolism is the basis of improving oil quality and increasing oil yield. The biochemical pathways of storage oil production in plants have been well documented. However, the factors regulating fatty acid synthesis and controlling total oil content in oilseed crops remain to be elucidated [30, 31]. According to our study, C16:0, C18:0 and C18:3n-3 were mainly synthesized at 5–15 DPA during the development of cottonseed. Synthesis of C18:2n-6 happened mainly in the middle and late stages of seed development. These fatty acid components showed an obvious correlation with each other during oil accumulation. In general, increase of C18:2n-6 was accompanied by decrease of other fatty acid components, particularly C18:3n-3, which decreased from 7.44% at 5 DPA to 0.02% at 60 DPA. Compared with their highest content at 15 DPA, C16:0 and C18:0 decreased by 50% and nearly 6 times in mature cottonseed, respectively. C18:1n-9 and C18:2n-6 accounted for more than 70% of all fatty acid components in mature cottonseed and were the main fatty acid components determining the total oil content of cottonseed.

The range of C18:1n-9 content in cottonseed is rather narrow among cotton germplasm resources [32], and it is difficult to breed high C18:1n-9 cotton varieties by conventional breeding methods [33]. Understanding the expression characteristics and functions of the key genes in fatty acid synthesis is the prerequisite for improving cottonseed oil quality. Expression of genes encoding key enzymes for fatty acid synthesis have been shown to be tissue and developmental stage specific [34]. We found that up-regulated and down-regulated DEGs were mainly observed at 15–40 DPA and 45–60 DPA, respectively (Fig. S2A), consistent with the results reported in a previous transcriptome sequencing experiment [22]. The relative content of most fatty acid components started to decrease after 30 DPA and C18:2n-6, the most abundant component, stabilized after 25 DPA (Table 1). In agree with this observation, down-regulated DEGs in the 30 DPA vs 20 DPA comparison were enriched with GO terms related to fatty acid biosynthesis (Table S12). Similar results have also been reported in soybean [35]. Through the analysis of GO enrichment and KEGG pathways of DEGs, it was found that fatty acid biosynthesis, fatty acid metabolism, and cutin and wax biosynthesis are involved in the early development of cottonseed. It has been proposed that *GhFAD2* expression increases to rapidly accumulate large amount of C18:2n-6, when the content of C16:0 and C18:0 exceeds a certain threshold [3]. We found that the expression level of *GhFAD2* increased significantly from 20 DPA (Fig. 1). Accordingly, the content of C18:2n-6 increased significantly from 20 DPA and reached its maximum at 40 DPA (Table 1). When the amount of substrate was lower than the threshold, the expression level of *GhFAD2* was down-regulated and tended to be relatively stable. And then, the very long chain fatty acid components and their derivatives, such as wax constituents, were synthesized in the seeds. Through the analysis of gene expression profile during cottonseed development, *GhFAD3* (*Gh_A09G0848* and *Gh_D09G0870*) was highly expressed at 5 DPA and decreased significantly at 20 DPA. After a further reduction at 25 DPA, *GhFAD3* maintained a very low expression level from 30 to 60 DPA. The expression profiles of *GhFAD2* and *GhFAD3* indicate these two enzymes play an important role in regulating the content of fatty acid components during cottonseed development.

Cotton has a complex allotetraploid genome and gene replication can lead to functional redundancy and diversification. It has been demonstrated that expression of ~ 30% of the cotton homoeologs are significantly A- or D-biased during fiber development [36]. We found that DEGs during the rapid oil accumulation period (10–30 DPA) are biased towards the D subgenome (Table S13). This was consistent with the result that high expression levels of fatty acid synthesis genes were found in the Dt homeologs [37]. Based on the analyses of WGCNA and the co-expression gene networks, the *SAD* genes (*Gh_A02G1788* and *Gh_D03G0588*) encoding the soluble desaturase enzyme that converts saturated C18:0 into unsaturated C18:1n-9 [38] were identified as the candidate hub genes highly associated with unsaturated fatty acid content at 25 DPA. *SAD* is highly active in developing cottonseeds, resulting in only 2–3% of C18:0 in the seed oil at maturity, but the C18:0 content can be significantly increased by silencing expression of *SAD* genes [10]. It is worth noting that the highest expression level of *SAD* (*Gh_A02G1788* and *Gh_D03G0588*) at 25 DPA was consistent with their preferential expression in developing ovules, especially at 25 DPA [39, 40]. The continuous decrease of C18:0 content from 15 to 25 DPA was accompanied by the increase of C18:1n-9 content, which is consistent with the result that *SAD* plays a decisive role in determining the ratio of saturated fatty acids to unsaturated fatty acids [38]. Moreover, the activity of *SAD* genes and consequently the C18:1n-9 content play important roles in senescence regulation, fungal infection resistance, and mechanical damage reactions [39, 41–44].

Because of its low specific gravity and high energy content, fat is the most economical and effective storage form of nutrients. During cottonseed development, the relative content of saturated C16:0 is always above 20%, indicating the importance of C16:0 in seed development and physiological activity. C16:0 is also a precursor for subsequent biosynthesis of other fatty acids. PUFAs are not only essential structural components of cells, but also play important roles in seed germination, physiological activity and resistance to adverse environment [3]. The content of PUFAs increased gradually during the period of seed maturation and the C18:2n-6 content was over 50% in mature cottonseed. C18:3n-3 is also an important substrate for biosynthesis of many other lipids and Jasmonic acid (JA) through the octadecanoid pathway. Although the content of C18:3n-3 was dramatically reduced from 20 DPA, it was thought that the threshold requirement for C18:3n-3 to synthesize JA is very low in cotton based on the observation of cotton anthers with only 1–2% of C18:3n-3 remaining fertile [25]. A high level of C18:2n-6 contributes to seed germination and early seedling growth under low temperature and salt conditions [45]. The highest content of PUFAs in cottonseed was C18:2n-6, not C18:3n-3. The ratio of C18:2n-6 to C18:3n-3 during cottonseed development was shown in Fig. S4. This accumulation pattern of fatty acid components in cottonseed is not accidental, but a result of nature selection closely related to the mechanisms of plants’ self-defense and protection of their offspring.

## Conclusions

Investigating the correlation between the content of various fatty acid components and the expression pattern of key genes responsible for fatty acid biosynthesis is a necessary step to understand the regulation of fatty acid metabolism and is also an effective way to explore the physiological significance of the key genes and the strategy for improving cottonseed oil quality. In this study, we have gained certain insights into the relationship between the dynamic changes of the expression levels of fatty acid biosynthesis genes and the accumulation of the main fatty acids in cottonseed, although the biological function of most DEGs is yet to be further investigated in future.

## Materials and methods

### Plant materials

Cotton (*G. hirsutum* L. cv Xinluzao 33) plants were grown in the field in Shihezi, China. Flowers were tagged on the day of blooming. Bolls were collected at 5, 10, 15, 20, 25, 30, 35, 40, 45, 50, 55, and 60 day post anthesis (DPA), respectively. No permissions were needed to study and collect these samples. Seeds were separated from bolls and stored in refrigerator at − 80 °C until further use. 5–60 DPA de-husked seeds were used in measurement of fatty acid content and transcriptome analysis.

### Fatty acid analysis

The content of fatty acid components of each sample was analyzed by external standard method. Thirty-seven fatty acid methyl esters (Sigma-Aldrich) with known concentration were diluted by 2, 5, 10, 20, 40 times with hexane (Table S1), respectively, and used to establish the quantitative standard curve of C14:0, C16:0, C18:0, C18:1n-9, C18:2n-6, and C18:3n-3 (Fig. S1). Seeds were ground into powder with liquid nitrogen, and then were freeze-dried using a vacuum freeze dryer. For each analysis, 1 g freeze-dried sample was transferred into glass tube, and oil was extracted using Soxtherm apparatus (Gerhadt). Then, 5 ml 0.4 M KOH-methanol and 5 ml hexane were added and mixed. The solution was transferred into vials and shaken for 30 min at 40 *°*C. Subsequently, the upper hexane layer was used in GC-MS analysis.

The qualitative and quantitative analyses were performed using GCMS-QP2020 (Shimadzu, Japan) at an electron ionization of 70 eV with an HP-88 column (100 m × 0.2 mm) and film thickness of 0.2 μm according to Liu et al. (2019) [25]. The details were as follows: after split/splitless injection at 250 °C and incubation in oven for 2 min at 40 °C, the temperature was increased to 240 °C at a rate of 4 °C/min and then held at 240 °C for 15 min. Helium was used as the carrier gas with a constant flow rate of 2 mL/min. A volume of 1.0 μL was injected and the injection mode was split with a ratio of 10:1. The ion source and interface temperature was set to 200 °C and 250 °C, respectively. The mass scan range was m/z 40–500 and the solvent delay time was set to 13 min. The quantification was carried out according to the response value of quantitative ions and the established standard curve. Each test was repeated three times, and the content of each fatty acid component was calculated as the percentage of total measured fatty acids.

### RNA isolation, library preparation and sequencing

Total RNA was extracted using Trizol reagent (Invitrogen) following the manufacturer’s protocol. RNA purity was checked using the NanoPhotometer® spectrophotometer (IMPLEN). RNA concentration was measured using Qubit® RNA Assay Kit in Qubit® 2.0 Flurometer (Life Technologies). RNA integrity was assessed using the RNA Nano 6000 Assay Kit with the Bioanalyzer 2100 system (Agilent). A total amount of 3 μg RNA per sample was used as input material for library preparation using NEBNext® Ultra™ RNA Library Prep Kit for Illumina® (NEB). The libraries were sequenced on an Illumina Hiseq platform. Using Hisat2 v2.0.5, paired-end clean reads were aligned to the TM-1 reference genome [23]. FeatureCounts v1.5.0-p3 was used to count the read numbers mapped to each gene. FPKM of each gene was calculated based on the length of the gene and the number of reads mapped to the gene.

### Differential expression, gene function annotation and enrichment analysis

Differential expression analysis was performed using the DESeq R package (1.18.0) through negative binomial distribution and calculation of false discovery rate [46]. The adjusted *p-value* < 0.05 and (|log_2_ (fold change) | ≥2 were used as the criteria for identification of DEGs among samples. Gene Ontology (GO) enrichment analysis of DEGs was implemented by the GO seq R package, in which gene length bias was corrected. GO terms with a corrected *p-value* less than 0.05 were considered to be significantly enriched. KOBAS software was used to test the statistical enrichment of DEGs in KEGG pathways.

### Quantitative RT-PCR validation of DEGs

Gene specific primers were designed using cDNA sequences of the target genes with the NCBI Primer-BLAST program (Table S2). qRT-PCR was carried out as described by Cheng et al. (2020) [47]. Three independent biological experiments were performed for samples of each time point. The relative expression levels were calculated with cotton ubiquitin gene (*GhUBI*, XM_012634824) as a reference according to Cheng et al. (2020) [47].

### Constructing co-expression networks of DEGs

The Weighted Correlation Network Analysis (WGCNA) package was used to construct the co-expression networks among DEGs associated with fatty acid synthesis in seeds from different developmental stages. The hub genes were screened on the basis of the module *K*_*ME*_ values and high-weight values. The correlation networks were drawn using Cytoscape 3.6.1.

## Supplementary Information

**Additional file 1: Fig. S1.** The quantitative standard curve and correlation coefficient of major fatty acid methyl esters in cottonseed determined by GC-MS. **Fig. S2.** The number and distribution of differentially expressed genes at 5–60 DPA. A, The number of DEGs that were up-regulated or down-regulated at 5–60 DPA. B, Venn diagram showing DEGs overlapping in different cottonseed development stages or unique to each developmental stage. **Fig. S3.** Heatmap comparison DEGs associated with cottonseed developmental stages. A,Venn diagram showed the different KEGGs in five stages. B and C showed the DEGs associated to the functional categories at 5, 10, 15, 20, 25 and 30 DPA respectively. B shows DEGs related to the biosynthesis of unsaturated fatty acids. Red represents high expression, and green represents low expression. Each row represents a DEG.S5, S10, S15, S20, S25and S30 represent 5, 10, 15, 20, 25and 30 DPA respectively. **Fig. S4.** The ratio of C18:2n-6 to C18:3n-3 during cottonseed development

**Additional file 2: Table S1.** Composition, retention times and fragment ions of the standard mixture of 37-component fatty acid methyl esters by GC-MS. **Table S2.** Primers used in qRT-PCR analysis.**Table S3.** Statistics of RNA-seq reads and mapping results.

**Additional file 3: Table S4.** Analysis of GO enrichment for 15,647 non-redundant DEGs

**Additional file 4: Table S5.** Analysis of GO enrichment from 20DPA to 30DPA

**Additional file 5: Table S6.** Analysis of GO enrichment from 35DPA to 60DPA

**Additional file 6: Table S7.** Analysis of KEGG enrichment for 15,647 common DEGs

**Additional file 7: Table S8.** Analysis of KEGG enrichment of the DEGs

**Additional file 8: Table S9.** Analysis of KEGG enrichment at the 35–60 DPA

**Additional file 9: Table S10.** Key KEGG pathways genes enriched in six module

**Additional file 10: Table S11.** Candidate hub genes in the brown module

**Additional file 11: Table S12.** Analysis of GO enrichment in down-regulated DEGs of the 30 DPA vs 20 DPA

**Additional file 12: Table S13.** Number of DEGs exhibiting homeolog expression bias during cotton seed development

## Data Availability

All Gene ID and annotation files could be obtained from CottonGen (https://www.cottongen.org). All other data generated or analyzed during this study are included in this manuscript.

## Abbreviations

DPA: Day Post-Anthesis
C14:0: Myristic acid
C16:0: Palmitic acid
C18:0: Stearic acid
C18:1n-9: Oleic acid
C18:2n-6: Linoleic acid
C18:3n-3: Linolenic acid
SFAs: Saturated fatty acids
PUFAs: Polyunsaturated fatty acids
MUFA: Monounsaturated fatty acid
ER: Endoplasmic reticulum
JA: Jasmonic acid
DEGs: Differentially expressed genes
qRT-PCR: Quantitative real-time polymerase chain reaction
RNA-seq: RNA-Sequencing
GO: Gene Ontology
KEGG: Kyoto Encyclopedia of Genes and Genomes
WGCNA: Weighted Correlation Network Analysis
*K*_*ME*_: Eigengene-based connectivity
FPKM: Fragments per kilobase of transcript per million mapped reads
