# Supplementary material for: Explore the gene network regulating the composition of fatty acids in cottonseed
Source: BMC Plant Biol. 2021 Apr 13;21:177. doi: 10.1186/s12870-021-02952-4 (PMC8042725; doi:10.1186/s12870-021-02952-4)
Supplement: Supplementary file 1 — Additional file 1: Fig. S1. The quantitative standard curve and correlation coefficient of major fatty acid methyl esters in cottonseed determined by GC-MS. Fig. S2. The number and distribution of differentially expressed genes at 5–60 DPA. A, The number of DEGs that were up-regulated or down-regulated at 5–60 DPA. B, Venn diagram showing DEGs overlapping in different cottonseed development stages or unique to each developmental stage. Fig. S3. Heatmap comparison DEGs associated with cottonseed developmental stages. A,Venn diagram showed the different KEGGs in five stages. B and C showed the DEGs associated to the functional categories at 5, 10, 15, 20, 25 and 30 DPA respectively. B shows DEGs related to the biosynthesis of unsaturated fatty acids. Red represents high expression, and green represents low expression. Each row represents a DEG.S5, S10, S15, S20, S25and S30 represent 5, 10, 15, 20, 25and 30 DPA respectively. Fig. S4. The ratio of C18:2n-6 to C18:3n-3 during cottonseed development [file 12870_2021_2952_MOESM1_ESM.docx]

**Explore the gene network regulating the composition of fatty acids in cottonseed**

Lihong Ma^1#^, Xinqi Cheng^1#^, Chuan Wang^1^, Xinyu Zhang^1^, Fei Xue^1^, Yanjun Li^1^, Qianhao Zhu^2^, Jie Sun^1^*, Feng Liu^1,^*

^1^Key Laboratory of Oasis Eco-agriculture, College of Agriculture, Shihezi University, Shihezi 832000, Xinjiang, China;

^2^CSIRO Agriculture and Food, GPO Box 1700, Canberra 2601, Australia;

# contributed equally

***Corresponding Authors:**

Feng Liu Email: [liufeng@shzu.edu.cn](mailto:liufeng@shzu.edu.cn;)

Jie Sun Email: sunjie@shzu.edu.cn

**
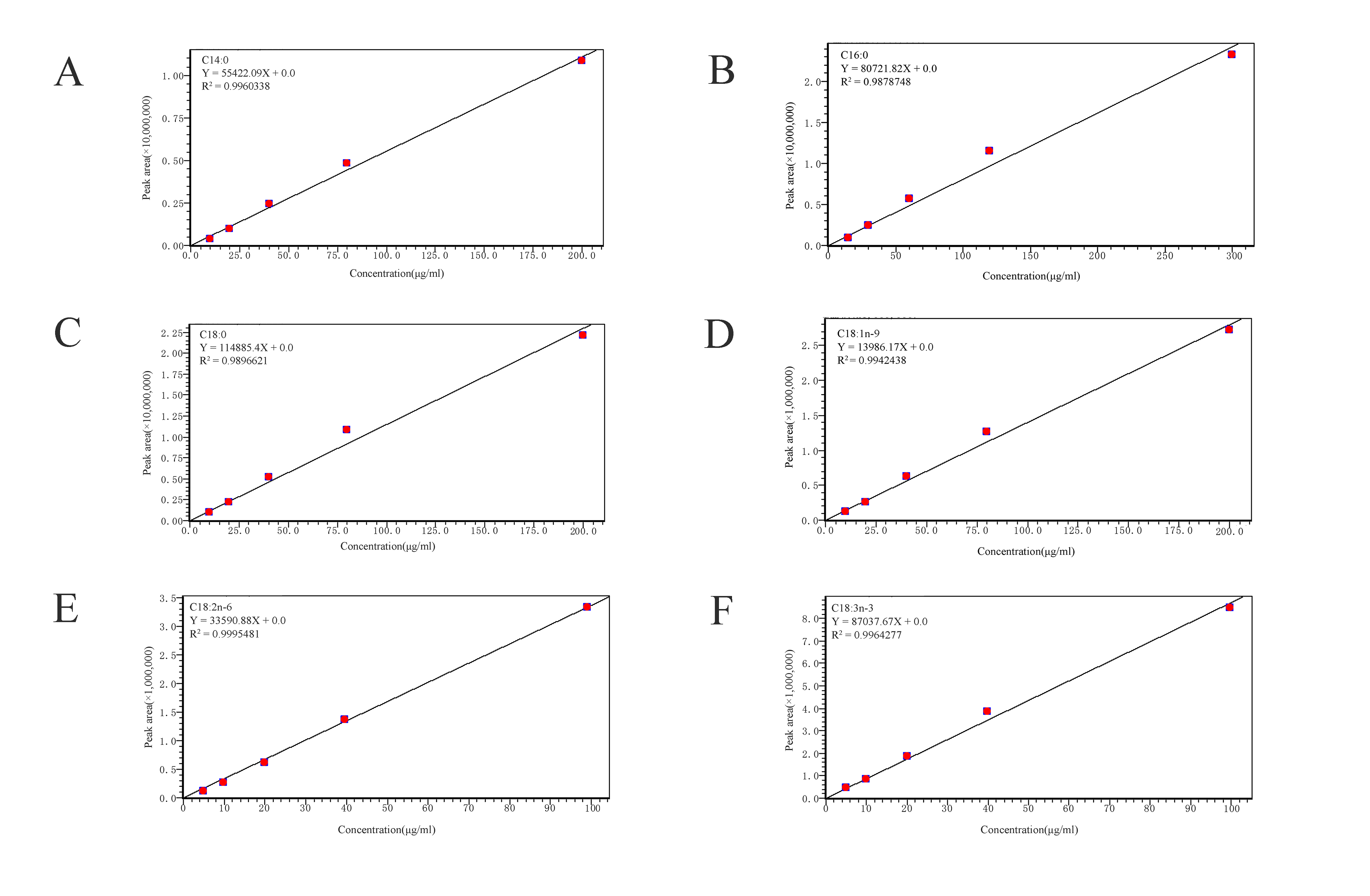
**

**Fig.S1** The quantitative standard curve and correlation coefficient of major fatty acid methyl esters in cottonseed determined by GC-MS.

C14:0, myristic acid; C16:0, palmitic acid; C18:0, stearic acid; C18:1n-9, oleic acid; C18:2n-6, 1inoleic acid; C18:3n-3, α-1inolenic acid.


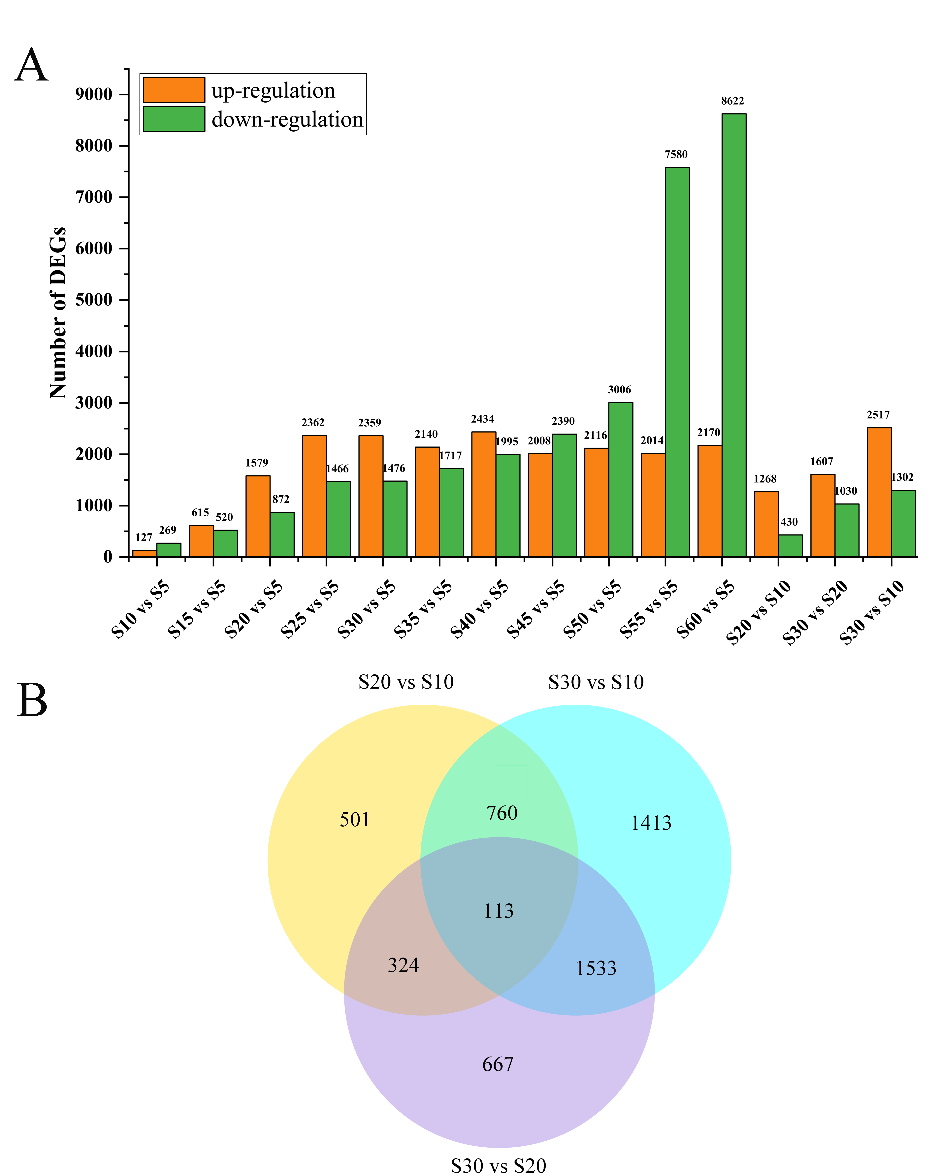


**Fig.S2** The number and distribution of differentially expressed genes at 5-60 DPA.

(A) The number of DEGs that were up-regulated or down-regulated at 5-60 DPA. (B) Venn diagram showing DEGs overlapping in different cottonseed development stages or unique to each developmental stage.

**
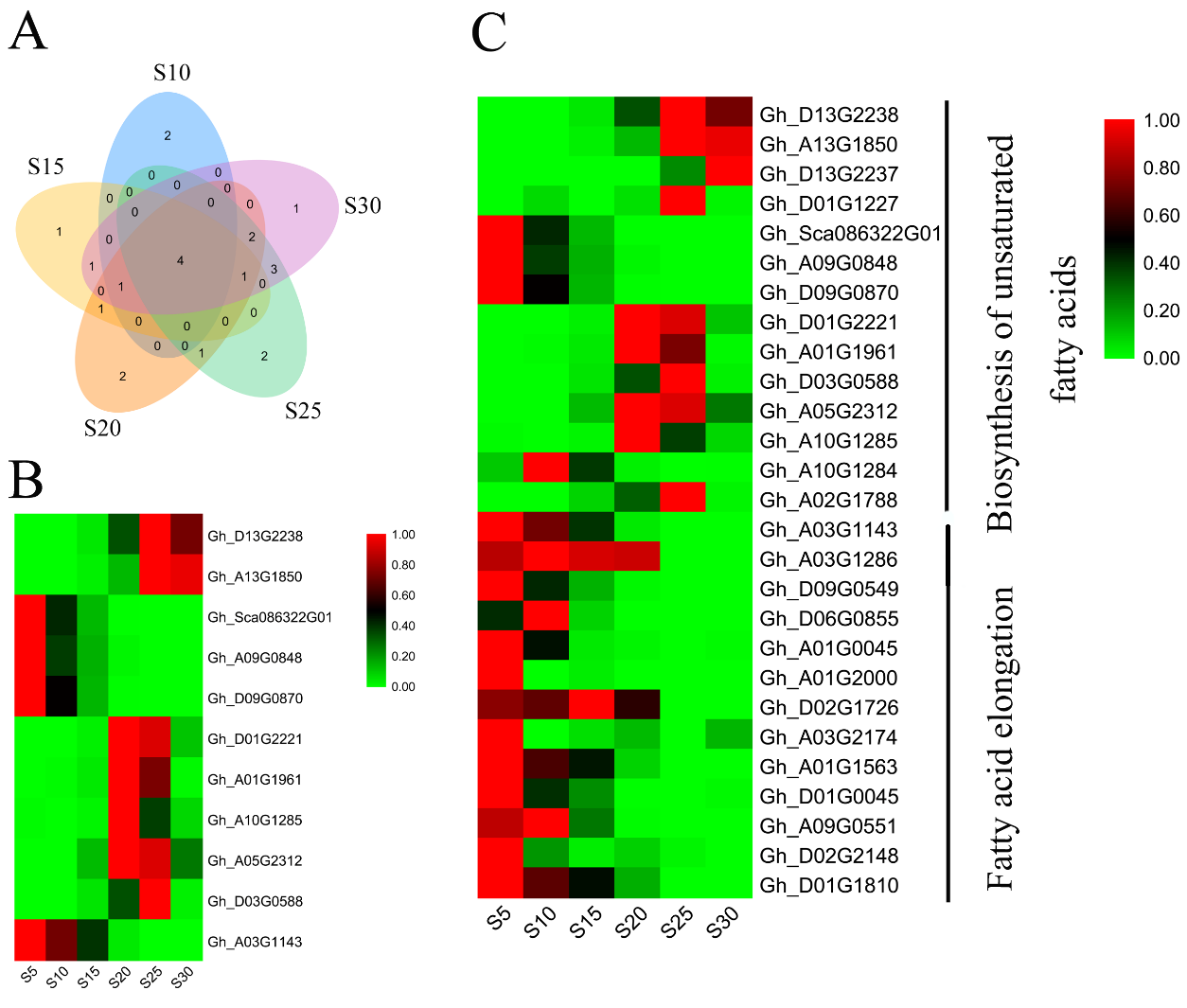
**

**Fig. S3** Heatmap comparison DEGs associated with cottonseed developmental stages.

(A) Venn diagram showed the different KEGGs in five stages. (B) and (C) DEGs associated to the functional categories at 5, 10, 15, 20, 25 and 30 DPA respectively. (B) DEGs related to the biosynthesis of unsaturated fatty acids. Red represents high expression, and green represents low expression. Each row represents a DEG.S5, S10, S15, S20, S25 and S30 represent 5, 10, 15, 20, 25 and 30 DPA respectively.

**
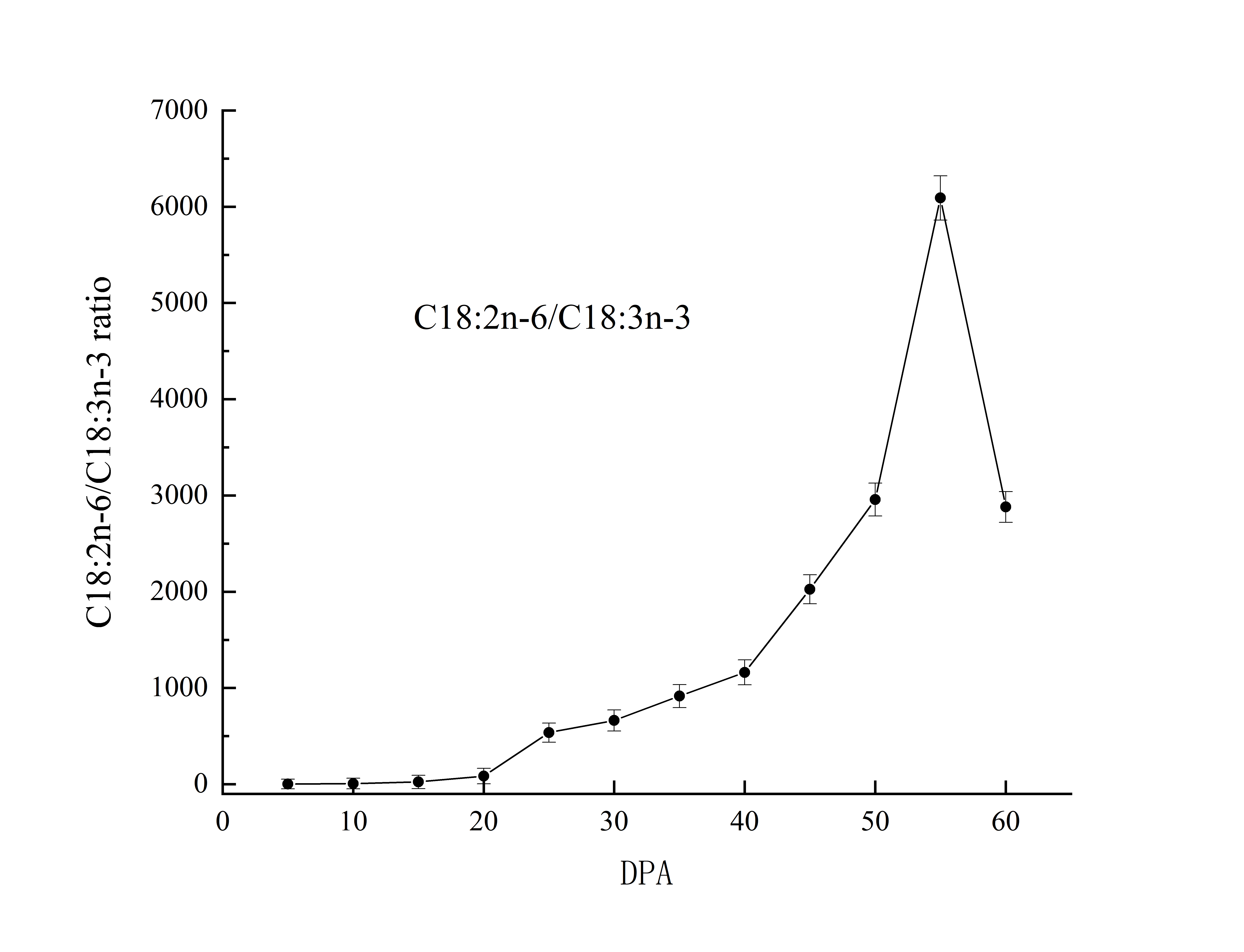
**

**Fig. S4** The ratio of C18:2n-6 to C18:3n-3 during cottonseed development
