# Supplementary material for: Explore the gene network regulating the composition of fatty acids in cottonseed
Source: BMC Plant Biol. 2021 Apr 13;21:177. doi: 10.1186/s12870-021-02952-4 (PMC8042725; doi:10.1186/s12870-021-02952-4)
Supplement: Supplementary file 2 — Additional file 2: Table S1. Composition, retention times and fragment ions of the standard mixture of 37-component fatty acid methyl esters by GC-MS. Table S2. Primers used in qRT-PCR analysis.Table S3. Statistics of RNA-seq reads and mapping results. [file 12870_2021_2952_MOESM2_ESM.docx]

Table S1 Composition, retention times and fragment ions of the standard mixture of 37-component fatty acid methyl esters by GC-MS

| No. | Compound | Standard concentration μg/mL | Retention times /min | m/z |
| --- | --- | --- | --- | --- |
| 1 | Methyl butanoate; C4:0 | 399.6 | 14.74 | 87 |
| 2 | Methyl caproate; C6:0 | 399.6 | 20.547 | 99 |
| 3 | Methyl caprylate; C8:0 | 399.8 | 26.326 | 127 |
| 4 | Methyl caprate; C10:0 | 399.8 | 31.57 | 155 |
| 5 | Methyl undecanoate; C11:0 | 199.8 | 33.966 | 169 |
| 6 | Methyl laurate; C12:0 | 399.7 | 36.22 | 214.2 |
| 7 | Methyl tridecanoate; C13:0 | 198.2 | 38.345 | 228.2 |
| 8 | Methyl myristate; C14:0 | 399.7 | 40.351 | 242.2 |
| 9 | Methyl myristoleate; C14:1n-5 | 199.8 | 41.752 | 240.2 |
| 10 | Methyl pentadecanoate; C15:0 | 199.6 | 42.249 | 256.2 |
| 11 | Methyl cis-10-pentadecenoate; C15:1n-5 | 199.8 | 43.614 | 222 |
| 12 | Methyl palmitate; C16:0 | 599.5 | 44.049 | 270.3 |
| 13 | Methyl palmitoleate; C16:1n-7 | 199.8 | 45.147 | 268.2 |
| 14 | Methyl heptadecanoate; C17:0 | 199.2 | 45.756 | 284.3 |
| 15 | Methyl cis-10-heptadecenoate; C17:1n-7 | 199.7 | 46.831 | 282.3 |
| 16 | Methyl stearate; C18:0 | 399.6 | 47.387 | 298.3 |
| 17 | Methyl elaidate; C18:1n-9t | 199.9 | 47.993 | 296.3 |
| 18 | Methyl oleate; C18:1n-9 | 399.6 | 48.303 | 296.3 |
| 19 | Methyl linolelaidate; C18:2n-6tt | 199.8 | 48.965 | 294.3 |
| 20 | Methyl linoleate; C18:2n-6 | 197.9 | 49.649 | 294.3 |
| 21 | Methyl arachidate; C20:0 | 399.7 | 50.426 | 326.3 |
| 22 | Methyl γ-linolenate; C18:3n-6 | 197.6 | 50.605 | 292.2 |
| 23 | methyl-α-linolenate; C18:3n-3 | 199.9 | 51.175 | 292.2 |
| 24 | Methyl cis-11-eicosenoate; C20:1n-9 | 199.1 | 51.3 | 292 |
| 25 | Methyl heneicosanoate; C21:0 | 199.2 | 51.845 | 340.3 |
| 26 | Methyl all-cis-11,14-eicosadienoate; C20:2n-6 | 199.8 | 52.592 | 322.3 |
| 27 | Methyl behenate; C22:0 | 398.7 | 53.247 | 354.3 |
| 28 | Methyl dihomo-γ-linolenate; C20:3n-6 | 199.9 | 53.541 | 320.3 |
| 29 | Methyl all-cis-11,14,17-Icosatrienoate; C20:3n-3 | 198.6 | 54.105 | 264 |
| 30 | Methyl erucate; C22:1n-9 | 199.8 | 54.158 | 278 |
| 31 | Methyl all-cis-5,8,11,14-Eicosatetraenoate; C20:4n-6 | 199.8 | 54.267 | 203 |
| 32 | Methyl tricosanoate; C23:0 | 199.1 | 54.668 | 368.4 |
| 33 | Methyl all-cis-13,16-Docosadienate; C22:2n-6 | 199.7 | 55.532 | 350.3 |
| 34 | Methyl all-cis-5,8,11,14,17-Eicosapentaenoate; C20:5n-3 | 199.9 | 55.923 | 201 |
| 35 | Methyl lignocerate; C24:0 | 399.6 | 56.148 | 382.4 |
| 36 | Methyl nervonate; C24:1n-9 | 196.8 | 57.179 | 380.4 |
| 37 | Methyl all-cis-4,7,10,13,16,19-Docosahexaenoate; C22:6n-3 | 199.7 | 60.384 | 173 |

**Table S2 Primers used in qRT-PCR analysis**

| Gene ID | Predicted coding protein | Primer sequence (5’-3’) | Product length (bp) |
| --- | --- | --- | --- |
| *Gh_A01G1574* | acetyl-CoA carboxylase | F: ATGTTAGAGGCCCAAAGGTC  R: GATCAGGAGCCCTGCTACAA | 94 |
| *Gh_D05G2554* | acetyl-CoA carboxylase | F: GATGAATTTTGTTCTGCACT  R: TAAATTTGACAGCTGCCATT | 85 |
| *Gh_D13G2493* | β-ketoacyl-ACP synthase II | F: ATGACATCTTCATCACTGGCAAGT  R: TTGAACAATGGAGACCGGGA | 102 |
| *Gh_A08G2201* | β-ketoacyl-ACP synthase II | F: TGGTAAGTCCACTATGTAATC  R: CATGGAGAAGAAATAAAAGC | 85 |
| *Gh_A08G1740* | acyl-ACP thioesterase A | F: GAAGTTTTCTTATTGCAATGCG  R: AACAGCTTGAATAGGCGTCA | 151 |
| *Gh_A06G0514* | acyl-ACP thioesterase B | F: TGCCACTGCTGCTACATCCT  R: CCAGAAGAAGCAGATGGTTT | 132 |
| *Gh_D02G1097* | stearoyl-ACP desaturase | F: TGTGCAATTCAATCCCATGG  R: GGTGGAAGAGTGGAGACCAT | 111 |
| *Gh_D05G3162* | stearoyl-ACP desaturase | F: TTGAATTTTAATGCCATCGC  R: AACTTGGGAGATCTAAGGGT | 86 |
| *Gh_D13G2238* | Fatty acid desaturase 2 | F: ATGGGATTGGTTGCGAGGAG  R: GTGGCATCGTTGAGAAGAGGTG | 119 |
| *Gh_D11G3169* | Fatty acid desaturase 2 | F: CGTCACAATCACCATTCAG  R: CGTTGTAGATAGGACCGTAT | 119 |
| *Gh_A09G0848* | Omega-3 fatty acid desaturase | F: CTGTAATCGGTCCATCCAT  R: GCTTCTGCTCGTATCCAT | 111 |
| *Gh_A07G0946* | Omega-3 fatty acid desaturase | F: GTTGAAAAGGGAGTATGTTA  R: ATTCTTGATCCAACACTGCT | 121 |

Gene IDs and protein annotations were based on Zhang et al. (2015).

**Table S3 Statistics of RNA-seq reads and mapping results**

| **Sample name** | **5DPA** | **10DPA** | **15DPA** | **20DPA** | **25DPA** | **30DPA** | **35DPA** | **40DPA** | **45DPA** | **50DPA** | **55DPA** | **60DPA** |
| --- | --- | --- | --- | --- | --- | --- | --- | --- | --- | --- | --- | --- |
| **Total reads** | 23,436,232 | 24,330,706 | 22,933,409 | 23,503,293 | 24,773,036 | 22,768,872 | 23,180,283 | 23,136,119 | 26,042,619 | 23,200,534 | 23,954,562 | 25,732,984 |
| **Total mapped** | 96.34% | 96.38% | 96.22% | 96.24% | 95.56% | 94.75% | 94.46% | 95.06% | 95.16% | 94.82% | 95.54% | 93.35% |
| **Multiply mapped** | 7.05% | 7.24% | 7.50% | 7.18% | 6.36% | 5.98% | 6.18% | 6.23% | 5.81% | 5.69% | 5.45% | 5.55% |
| **Uniquely mapped** | 89.29% | 89.14% | 88.72% | 89.06% | 89.20% | 88.77% | 88.29% | 88.84% | 89.36% | 89.14% | 90.09% | 87.80% |
| **Read-1** | 44.82% | 44.73% | 44.52% | 44.70% | 44.91% | 44.73% | 44.40% | 44.74% | 45.06% | 44.98% | 45.30% | 44.23% |
| **Read-2** | 44.47% | 44.41% | 44.20% | 44.36% | 44.30% | 44.04% | 43.89% | 44.10% | 44.30% | 44.16% | 44.79% | 43.57% |
| **Reads map to '+'** | 44.62% | 44.54% | 44.34% | 44.50% | 44.60% | 44.42% | 44.20% | 44.45% | 44.71% | 44.56% | 45.01% | 43.85% |
| **Reads map to '-'** | 44.68% | 44.60% | 44.38% | 44.56% | 44.61% | 44.35% | 44.09% | 44.39% | 44.65% | 44.58% | 45.09% | 43.95% |
| **Non-spliced reads** | 54.95% | 54.68% | 54.06% | 54.71% | 56.01% | 60.79% | 60.55% | 61.59% | 60.66% | 59.65% | 59.18% | 55.78% |
| **Spliced reads** | 34.35% | 34.46% | 34.66% | 34.35% | 33.20% | 27.98% | 27.74% | 27.25% | 28.70% | 29.50% | 30.91% | 32.02% |

Total reads: The total number of clean reads after filtering out low quality reads; Total mapped: the percentage of the total number of reads mapped to the reference genome; Multiply mapped: the percentage of reads that have multiple alignment positions on the reference sequence; Uniquely mapped: the percentage of reads that have unique alignment positions on the reference sequence; Reads map to'+' and Reads map to'-': the percentage of reads alignment to the positive and negative strands of the genome, respectively; Spliced reads: the percentage of aligned reads that are across the junction of two exons; Non-spliced reads: the percentage of reads that are fully aligned to exons and without across junction of two exons.
